# Association Between Antibiotic Exposure and the Risk of Male Infertility: A Case–Control Study

**DOI:** 10.3390/jox15050172

**Published:** 2025-10-21

**Authors:** Friday E. Okonofua, Lorretta Favour C. Ntoimo, Titus A. M. Msagati, Oladiran Ayodeji, Michael Aziken, Akhere Omonkhua, Victor Ohenhen, Celestina Olafusi, Moses O. Alfred

**Affiliations:** 1 Department of Obstetrics and Gynaecology, University of Benin Teaching Hospital, University of Benin, Benin City 300213, Nigeria; michael.aziken@uniben.edu; 2 Women’s Health and Action Research Centre (WHARC), Benin City 300283, Nigeria; lorretta.ntoimo@fuoye.edu.ng; 3 Department of Demography and Social Statistics, Federal University Oye-Ekiti, Oye-Ekiti 371104, Nigeria; 4 Institute for Nanotechnology and Water Sustainability, College of Science, Engineering and Technology, The Science Campus, University of South Africa, Corner Pioneer Avenue and Christiaan De Wet, Roodepoort, Johannesburg 1709, South Africa; msagatam@unisa.ac.za; 5 Department of Obstetrics and Gynaecology, University of Medical Sciences, Ilaje, Ondo 351104, Nigeria; ladiran54@gmail.com; 6 Department of Medical Biochemistry, School of Basic Medical Sciences, University of Benin, Benin City 300213, Nigeria; akuekegbe.omonkhua@uniben.edu; 7 Department of Obstetrics and Gynaecology, Central Hospital, Benin City 300102, Nigeria; victorohenhen@gmail.com; 8 Department of Biochemistry, University of Medical Sciences, Ilaje, Ondo 351104, Nigeria; olafusicelestina@gmail.com; 9 Department of Chemical Sciences, Redeemer’s University, Ede 232101, Nigeria; alfredm@run.edu.ng; 10 African Centre of Excellence for Water and Environmental Research (ACEWATER), Redeemer’s University, Ede 232101, Nigeria

**Keywords:** male infertility, urinary antibiotics, Nigeria, case–control study, sperm quality

## Abstract

Irregular use of antibiotics is widespread in Nigeria, which has been reported to be associated with the prevalence of antibiotic resistance. As antibiotics have been reported to be associated with declining male infertility in animal models, we investigated the association between exposure to antibiotics and sperm quality. The study was a prospective case–control study involving 136 infertile men and 154 fertile men recruited from five hospitals in southern Nigeria. Semen analysis was carried out, while Liquid Chromatography Mass Spectrometry was used to assay for various antibiotics in urinary samples. Three antibiotics showed an independent association with sperm quality in the regression analysis. Urinary ampicillin was associated with more than a 3 mL decline in sperm count in the cases, with no significant effects shown in the control group. Similarly, a significant association of urinary chloramphenicol with sperm motility and sperm morphology was shown in cases of infertility. In contrast, an independent association of urinary tetracycline with increased sperm motility in men with infertility was found, but no significant association was shown in fertile men. We conclude that urinary antibiotics—ampicillin, tetracycline, and chloramphenicol—may be associated with varying sperm characteristics and sperm quality in Nigerian men.

## 1. Introduction

Despite the rising prevalence of male factor infertility in many African countries [1,2], it is regrettable that not much has been achieved in identifying its root causes, which are necessary to identify effective solutions. While exposures to environmental contaminants have been put forward as important risk factors [3,4,5], the full range of toxins and contaminants that adversely disrupt sperm count and quality has not been identified.

In our previous research involving a similar group of fertile and infertile Nigerian men, we found that higher levels of mycotoxin and paraben metabolites in urine are associated with poor sperm quality and survival. While we identified some associations between exposure to parabens and mycotoxins and male infertility, we believed it was essential to further explore the relationship between male infertility and the use of antibiotics, which are commonly prescribed and used in our population.

Antibiotics are another group of agents commonly used for the treatment of genital and non-genital infections, with the possibility that they could influence the quality of spermatozoa. A systematic review and meta-analysis of published studies on the effect of antibiotics on male infertility [6] concluded that antibiotic treatment of men with increased spermatozoa leucocytes improved the quality of sperm ejaculates.

Genital tract infection is often thought to be a cause of male infertility in Nigeria, for which antibiotics are prescribed, whether or not there is clear evidence of infection [7,8,9]. To date, the extent to which such treatments influence the probability of male infertility or fertility is not yet established. Whereas antibiotics prescribed for genital tract infections are essential for treating reproductive pathogens, improper use of antibiotics in situations where infections do not exist or where the causative organism has not been identified can lead to the emergence of resistance strains of bacteria, which could worsen the semen quality and characteristics [10].

In our previous investigation and analysis of urinary samples of fertile and infertile men in southwest Nigeria, we identified a large number of samples with high levels of different types of antibiotics [11,12]. We speculated the possibility of these antibiotics being independently associated with male infertility of fertility after controlling for possible confounding variables. We were particularly interested in investigating this relationship, as it could provide a simple method for counselling couples and designing interventions for reducing the high prevalence rate of infertility in Nigerian men. Therefore, the objective of the study was to identify the types of antibiotics and their metabolites in urinary samples of fertile compared with infertile Nigerian men and to determine their association with male infertility. We believe the results will help the ongoing debate regarding the rational and appropriate use of antibiotics in the country.

## 2. Materials and Methods

### 2.1. Study Design and Population

The study was designed as a case–control study that compared urinary levels of antibiotics between men with low sperm count and poor-quality spermatozoa with men with normal sperm count and quality. This report was part of a study that investigated the effects of various environmental contaminants and toxins in association with male infertility in Nigerian men, the design and some of the findings of which have been reported elsewhere [11,12].

### 2.2. Sample Size

The sample size was 290 men aged 18–65 years, consisting of 136 infertile men (cases) and 154 fertile men (controls). The sample size determination and distribution by the health facilities have been published elsewhere [11]. The study participants were recruited from two tertiary hospitals [University of Medical Sciences Teaching Hospital (UNIMEDTH), and the University of Benin Teaching Hospital (UBTH)]; one secondary hospital (Central Hospital, Benin City, Nigeria), and two private hospitals that provide tertiary infertility treatment (Abel Guobadia Specialist Hospital, and the Graceland Specialist Hospital, both in Benin City. Apart from UNIMEDTH, which is located in Ondo, about 200 km from Benin City, three of the four hospitals in Benin are located within 2 km of each other.

### 2.3. Data Collection

Research teams at various hospitals, coordinated by Abel Guobadia Hospital, recruited participants for an infertility study. Participants were informed about the study during their first clinic visit and provided urine and blood samples if they agreed to participate. The protocol covered socio-demographics, reproductive health history, exposures, behaviours, and semen and hormone analysis. Urine samples were filtered and stored at −40 °C for later analysis, while semen samples were analysed immediately. Details of how the participants were characterised as fertile and infertile, recruitment, data collection, sample preparation, semen analysis, quality assurance, and hormone assay are published elsewhere [11,12].

### 2.4. Analysis of Urinary Antibiotics

The biochemical analysis of antibiotic metabolites from urine samples was conducted for 179 respondents whose samples were available using Liquid Chromatography Mass Spectrometry/Mass Spectrometry (LCMS/MS) in the Biochemistry Department at the University of South Africa (UNISA). There is no statistically significant difference in the characteristics of the respondents whose urine underwent biochemical analysis and those whose urine did not (N = 111), except in their religious affiliation [11]. The details of the analytical method have been reported elsewhere [11,12].

### 2.5. Data Analysis

The sociodemographic characteristics of the study population are presented in frequencies and percentages, while summary statistics were used for the characteristics that are continuous variables, including sperm parameters and antibiotics. The mean with standard deviation was used for variables that were normally distributed, and the median with interquartile range was used when the variables were not normally distributed. To test the significant difference between the control and cases, the independent samples t-test was used when the variables were normally distributed, and the non-parametric alternative (Mann–Whitney test) was used when the assumption of normality was violated. None of the sperm parameters or the antibiotics were normally distributed. A non-parametric regression was used to estimate the effect of the antibiotics on the sperm parameters. The analysis was conducted using Stata 17 npregress kernel. The variables of interest (antibiotics) were modelled nonparametrically through kernel smoothing based on the Nadaraya-Wastson estimator, with bandwidths selected using the normal-reference rule of thumb and the Epanechnikov kernel was used. Two models were estimated for each sperm parameter and antibiotic. The unadjusted model contained only the specific sperm parameter outcome variable and one antibiotic. Due to the small sample size, the adjusted model controlled for the effect of key variables identified to influence sperm quality in the study population, namely, age, body mass index, type of occupation, and testosterone. These additional covariates in the adjusted model were included parametrically (linearly). Stata 17 for Windows was used to conduct all the analyses. All the analyses were two-tailed, the statistical significance level was 0.05, and standard errors were estimated using the bootstrap method.

## 3. Results

### 3.1. Socio-Demographic Characteristics of the Study Population

The socio-demographic characteristics of the 179 respondents whose urine underwent the biochemical analysis of the metabolites of antibiotics are presented in Table 1. Their age ranged from 18 to 64 years, with a mean age of 40.9 (SD 7.6) for the cases and 40.1 (SD 7.9) for the control group. The mean weight was 80.8 kg (SD 14.8), 80.2 kg for the cases and 81.5 kg for the controls. The mean height was 1.7 m (SD 0.13) for the control group and 1.6 m (SD 0.11) for the cases. The majority were overweight and obese, with a mean body mass index of 26.7 (SD 5.4) for the cases and 27.6 (SD 5.1) for the control group. The majority were married and attained tertiary education. Close to 30% are engaged in diverse businesses as their occupation. Slightly above 10% drink alcohol often, whereas 44.8% were occasional alcohol drinkers. Only 3% smoked often, and 4.8% smoked occasionally. None of these characteristics was significantly different between the two groups.

### 3.2. Descriptive Analysis of the Sperm Parameters by Group

A descriptive analysis of the sperm parameters by fertility status is presented in Table 2. The median sperm count for the cases was 6(IQR 14), whereas it was 46(IQR 26.5) for the control group, and the difference was statistically significant. Active motility differed significantly between the cases and control, with a median of 10(IQR 24) for the cases and 40(IQR 19.5) for the control group. In total motility, there was a statistically significant variation between the two groups, with a median of 20(IQR 36) for the cases and 60(IQR 15.5) for the controls. The median morphology was significantly different between the cases 20(IQR 40) and the control group 57(IQR 20). Sperm volume differed significantly between the two groups. The median sperm volume for the cases was 2.2(IQR 1), and 2.6(IQR 1.5) for the control group.

### 3.3. Descriptive Analysis of Antibiotics by Group

Table 3 presents a description of the antibiotics by the fertility status of the men. The median level of ampicillin in the case and control groups was similar 0(IQR 0), but the range differed; in the cases, it was 0–47 and 0–223 in the control group. Metronidazole level was similar for both groups, with a higher IQR and upper limit in the range for the cases than the control. The median level of tetracycline was the same in both groups, with a lower IQR in the cases (28) than in the control group (51.5), and the range among the cases is higher than in the control group. The median level of chloramphenicol was similar between the groups, with a higher range in the cases. The median level of ciprofloxacin in the cases was 11(IQR 199.47), and 16(IQR 146.6) in the control group; the range is larger in the cases than in the controls. There was no statistically significant difference between the case and control groups in any of the antibiotics.

### 3.4. Distribution of Antibiotics by Sperm Parameters

**Figure A1:**
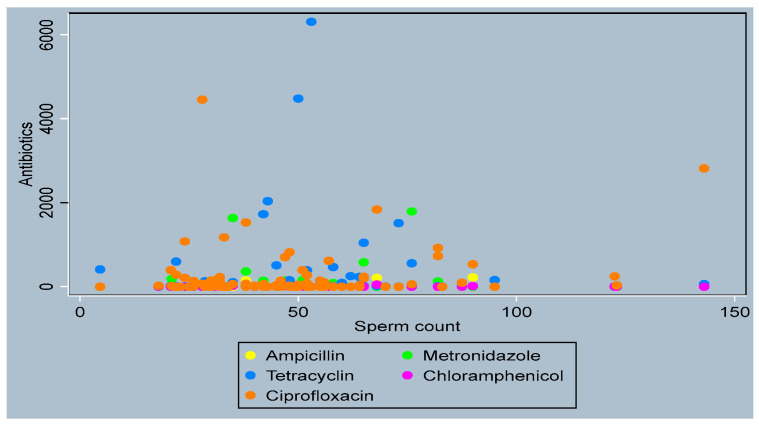
Distribution of antibiotics by sperm count in the controls.

**Figure A2:**
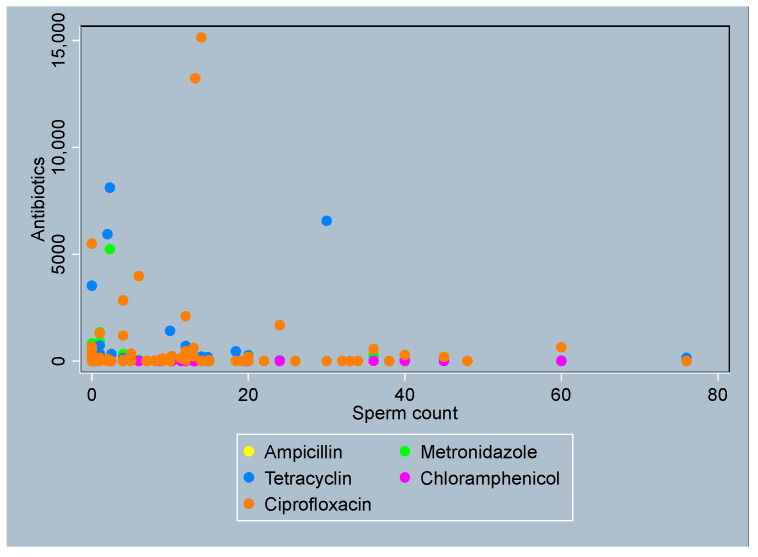
Distribution of antibiotics by sperm count in the cases.

**Figure A3:**
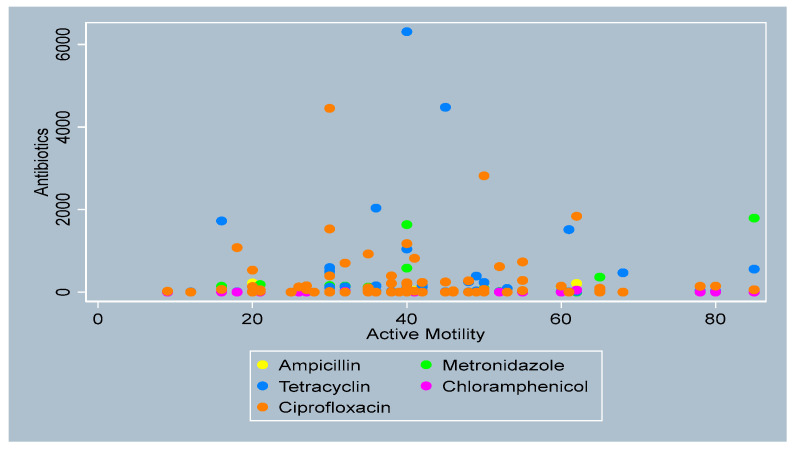
Distribution of antibiotics by active motility in the controls.

**Figure A4:**
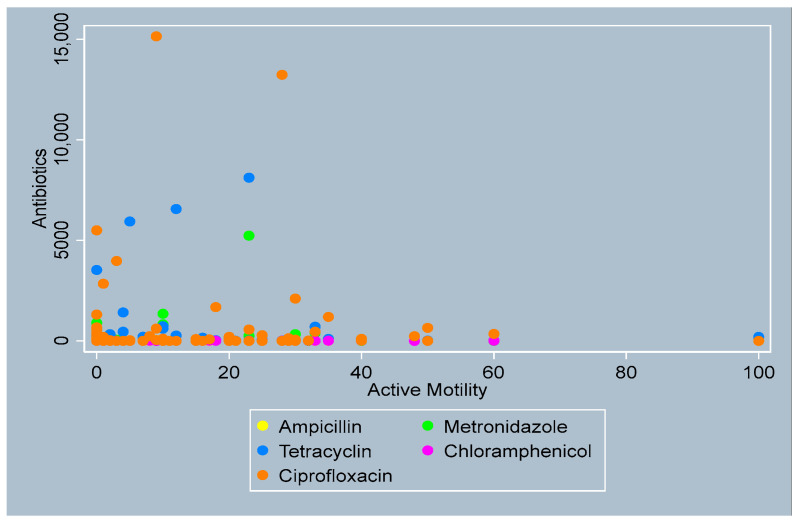
Distribution of antibiotics by active motility in the cases.

**Figure A5:**
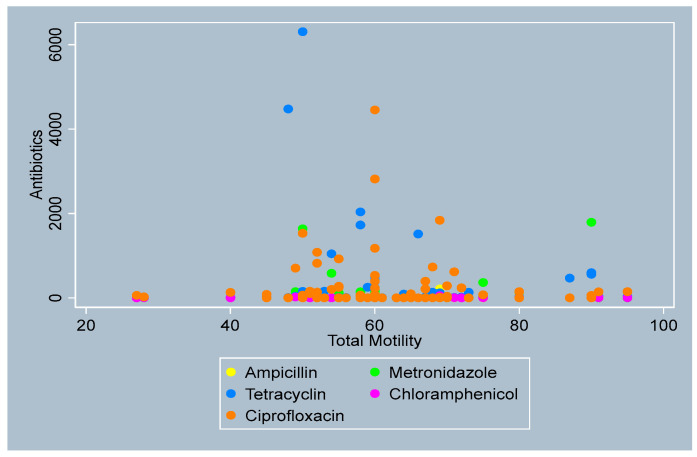
Distribution of antibiotics by total motility fin the control group.

**Figure A6:**
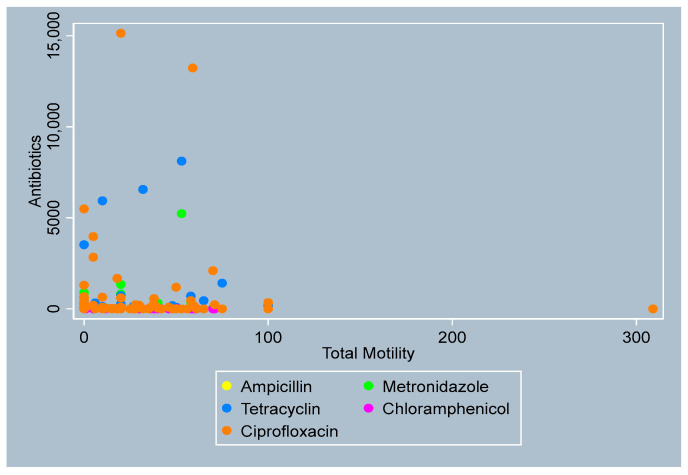
Distribution of antibiotics by total motility in the cases.

**Figure A7:**
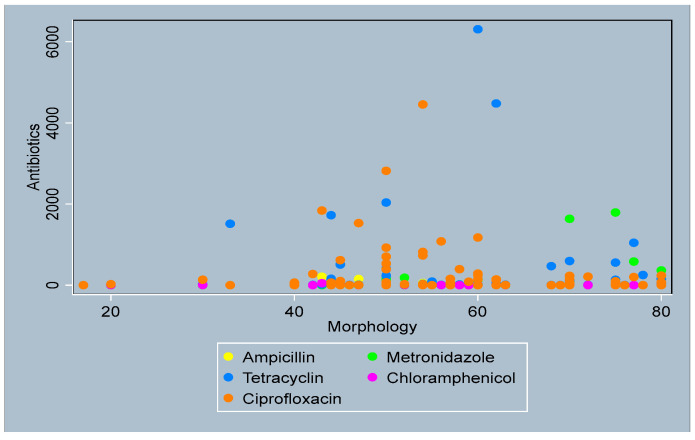
Distribution of antibiotics by morphology in the control group.

**Figure A8:**
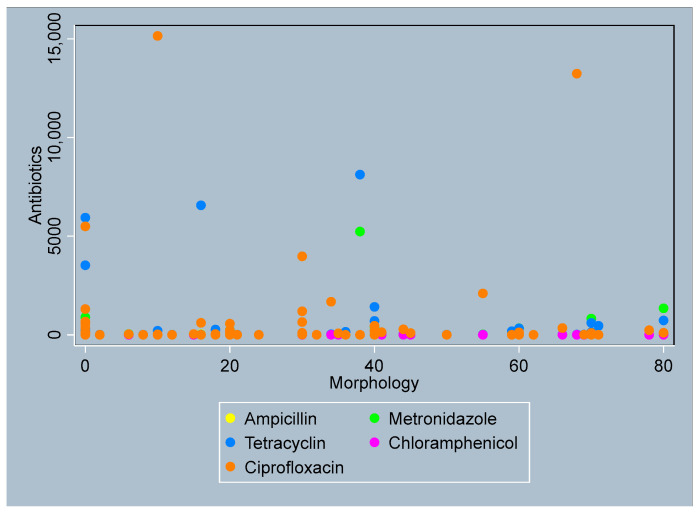
Distribution of antibiotics by morphology in the cases.

**Figure A9:**
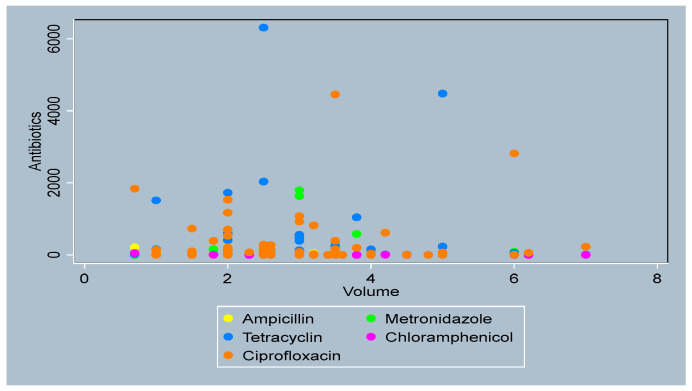
Distribution of antibiotics by volume in the control group.

**Figure A10:**
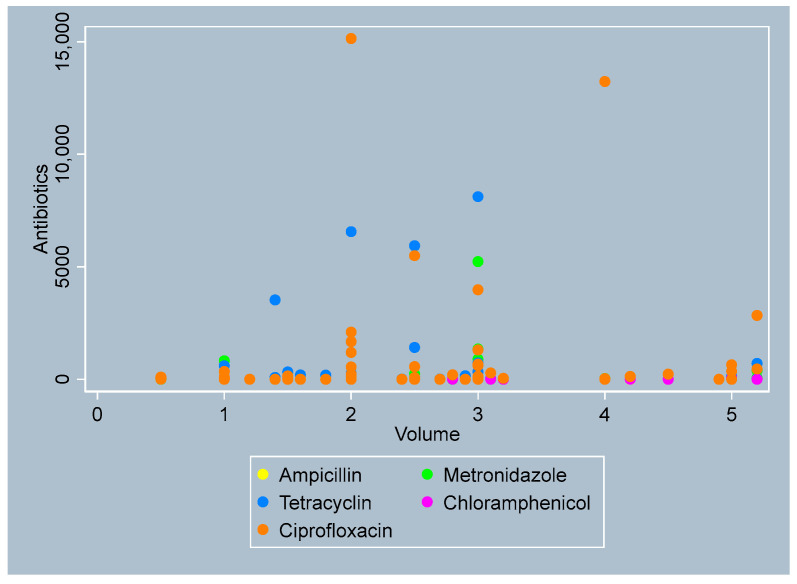
Distribution of antibiotics by volume in the cases.

The distribution of the antibiotics by each sperm parameter for the control and case groups is presented using graphs (Figure A1, Figure A2, Figure A3, Figure A4, Figure A5, Figure A6, Figure A7, Figure A8, Figure A9 and Figure A10) in Appendix A. Analysis of the distribution of the antibiotics by the semen parameters showed consistent differences between the control and case groups. Sperm counts were generally higher among controls, whereas cases exhibited a downward shift in distribution, indicating reduced spermatogenesis (Figure A1 and Figure A2). Figure A3, Figure A4, Figure A5 and Figure A6 demonstrate that both active and total motility were better in the control group, with cases clustering at lower motility ranges, reflecting impaired sperm movement. The distribution of morphology (Figure A7 and Figure A8) revealed that controls had a higher proportion of sperm with normal forms, whereas cases displayed a predominance of abnormal morphology. In Figure A9 and Figure A10, semen volume was higher in controls, but the difference between groups was less marked than for motility and morphology. These patterns indicate statistically significant group differences across most of the sperm parameters.

### 3.5. Multivariable Analysis of the Marginal Effect of Antibiotics on Sperm Parameters

The results of the unadjusted and adjusted models of the non-parametric regression analysis estimating the marginal effect of the antibiotics on sperm parameters are presented in Table 4. Holding the selected demographics and testosterone level constant, a unit increase in ampicillin among the cases significantly decreased sperm count by 3.455. Ampicillin also decreased sperm count in the control group, but it is statistically insignificant.

A unit increase in ampicillin significantly increased active motility in the cases in the unadjusted model. However, when the demographics and testosterone were adjusted, the effect became statistically insignificant and inverse.

Active motility decreased insignificantly among the cases with increasing levels of tetracycline in the unadjusted model, but when other factors were controlled, the effect became statistically significant and positive—an increase in the level of tetracycline increased active motility by 0.081. A similar pattern was observed for tetracycline in total motility among the cases; one unit increase in tetracycline significantly increased total motility by 0.068 in the adjusted model. An increase in the level of tetracycline in the control group also increased total motility significantly, but only in the unadjusted model.

An increase in chloramphenicol level significantly decreased total motility in the cases. The decreasing effect was not statistically significant in the unadjusted model, but it became statistically significant when other factors were controlled, reducing total motility by 3.994.

An increase in the level of ampicillin increased morphology in the cases by 2.727 in the unadjusted model; the direction of effect remained positive but statistically insignificant when other factors were adjusted. The sperm morphology in the control group was significantly affected by the level of chloramphenicol. One unit increase in chloramphenicol significantly decreased morphology in the control group in both the adjusted and unadjusted models.

## 4. Discussion

The objective of this study was to determine the association of urinary levels of antibiotics with male infertility in a cohort of fertile and infertile men, not known to have taken recent antibiotics for the treatment of infections. The results showed different types of antibiotics—ampicillin, metronidazole, tetracycline, chloramphenicol, and ciprofloxacin—in urinary samples in both cases and controls. However, there was no difference between the cases of fertile and infertile men in the levels of urinary antibiotics, which indicates that both groups of men are equally exposed to routine antibiotics in this cohort. It also suggests that antibiotic exposure may not be a direct aggregated route cause of male infertility in this population. However, we investigated the association of the different antibiotics with various components of sperm parameters. Only ampicillin, tetracycline, and chloramphenicol showed a significant association with various aspects of sperm quality.

With respect to sperm count, urinary ampicillin was associated with more than a 3 mL decline in sperm count in the cases, with no significant effects in the control group. Other antibiotics did not show any significant association with sperm count. The result is consistent with several publications that show various toxicological effects of ampicillin and similar antibiotics on sperm count and sperm morphological characteristics in different animal models [7,8,9,13]. However, no such effects had previously been demonstrated in humans; this being the first study to report an association of urinary ampicillin with declining sperm count. Adesanya and Awobayo [14] reported that a 14-day administration of ampicillin, cloxacillin, and tetracycline significantly reduced the concentration of testosterone in male albino rats compared to controls. This suggests that the modulation of the hormone pathway responsible for the synthesis and maturation of spermatozoa may be the mechanism through which ampicillin may be associated with a reduction in sperm count in humans.

The results of this study show an independent association of urinary tetracycline with increased sperm motility in men with infertility, but no significant association was shown in fertile men. However, this is in contrast to several studies that show a decrease in sperm motility when tetracycline is administered to male rats [13,15]. Additionally, a study in humans [16] showed a dose-related inhibition by tetracycline in percent rapidly moving spermatozoa, and in higher doses, the spermatozoa became completely static. Our results showing the association of urinary tetracycline with increased sperm motility in men with infertility may suggest specific therapeutic effects of tetracycline in men experiencing infertility. Genital tract infections in men are largely associated with male infertility [4,17], it is possible that tetracycline may overwhelm the growth of specific bacteria responsible for sperm motility in infertile men. Further studies are required to exemplify this relationship in male infertility.

Further, the results of this study demonstrated a significant association of urinary chloramphenicol with sperm motility in the cases, and sperm morphology in the control group. Increasing levels of chloramphenicol showed a nearly 4-unit significant decrease in sperm motility and a 2-unit decline in sperm morphology. While no such relationship has been shown in humans, animal models reveal that chloramphenicol is a mitochondrial activity inhibitor, and its in vitro administration decreases mitochondrial protein synthesis, mitochondrial activity, and ATP level in sperm, and consequently reduces sperm linear motility speed, but not the motility. No such relationship has been shown in humans [18], Prolonged administration of chloramphenicol has also been shown to result in decreased sperm quality and male infertility in Wistar rats [19]. However, since other studies have reported that chloramphenicol may be associated with increases in sperm motility [20,21], it is important to investigate this relationship through further research to determine the effects of chloramphenicol on sperm characteristics in humans.

In sum, the results of our study, being one of the few studies in humans that investigate the association of various antibiotics with human sperm characteristics, indicate that ampicillin, tetracycline, and chloramphenicol may be associated with various sperm characteristics. However, the other antibiotics found in urinary samples—metronidazole and ciprofloxacin—did not show any association with sperm characteristics.

Nigeria is a country where individuals take antibiotics without prescriptions, and even for conditions that are not related to infection [22,23]. The high level of antibiotics observed in both fertile and infertile men in this study further demonstrates this tendency. The injudicious use of antibiotics in Nigeria has been cited as the reason for the high prevalence of antibiotic resistance in the country [24,25], which may partly explain the phenomenon observed in this study.

This study has both strengths and weaknesses. The major strength lies in its being one of the few such studies in humans that investigate the relationship between urinary antibiotics and sperm quality. Our use of high-fidelity Liquid Chromatography Mass Spectrometry/Mass Spectrometry to measure urinary levels of antibiotics also adds to the strength and novelty of the study. The limitation of the study is the use of urinary samples rather than blood samples, with blood samples more likely to show a wider range of antibiotics, and possibly a stronger association with sperm quality.

In the adjusted nonparametric models, the initially significant associations between chloramphenicol and total sperm motility among the cases, as well as between chloramphenicol and sperm morphology among the controls, became statistically insignificant after applying the Benjamini–Hochberg FDR correction. This change suggests that the observed associations in the analyses without the FDR correction may partly reflect chance findings arising from multiple comparisons. The attenuation of statistical significance following FDR adjustment underscores the need for cautious interpretation and supports the use of multiplicity correction to identify only the most robust antibiotic–sperm quality relationships. Nevertheless, as this study demonstrates a hitherto unknown relationship, we believe it will stimulate more research to investigate the use of antibiotics and their association with the rising rate of poor sperm characteristics in the country. It will also engender specific programs, policies, and clinical practices to influence the more judicious use of antibiotics in the country.

## 5. Conclusions

We conclude that urinary antibiotics—ampicillin, tetracycline, and chloramphenicol—may be associated with diverse sperm characteristics and sperm quality in Nigerian men. Further studies are required to identify the direction of these relationships and the full array of antibiotics that may be involved.

## Figures and Tables

**Table 1:** Characteristics of the study population.

Characteristic	Fertile N = 92	Infertile N = 87	Total N = 179	*p*-Value
	Mean(SD) [Range]	Mean(SD) [Range]	Mean(SD) [Range]	
**Age**	40.9(7.6) [20–62]	40.1(7.9) [18–64]	40.5(7.7) [18–64]	0.4823
**Weight(kg) (n = 177)**	80.2(15.2) [46–115]	81.5(14.5) [51.2–122]	80.8(14.8) [46–122]	0.5661
**BMI (n = 176)**	26.7(5.4) [16–41.2]	27.6(5.1) [16.2–43]	27.2(5.3) [16–43]	0.2698
**Height (meter) (n = 176)**	1.7(0.13) [1.3–1.9]	1.6(0.11) [1.3–1.9]	1.6(0.12) [1.3–1.9]	0.8599
	**No.(%)**	**No.(%)**	**No.(%)**	
**BMI (n = 176)**				0.202
Normal	28(31.1)	26(30.2)	54(30.7)	
Thin	6(6.7)	1(1.2)	7(4.0)	
Overweight	32(35.6)	28(32.6)	60(34.1)	
Obese	24(26.7)	31(36)	55(31.3)	
**Marital Status**				0.878
Single	8(9.0)	7(8.3)	15(8.7)	
Married	81(91.0)	77(91.7)	158(91.3)	
**Religion**				0.725
Islam	6(6.5)	4(4.6)	10(5.6)	
Christian	85(92.4)	81(93.1)	166(92.7)	
Traditional/Other	1(1.1)	2(2.3)	3(1.7)	
**Education**				0.808
Primary	7(7.6)	4(4.6)	11(6.1)	
Secondary	21(22.8)	24(27.6)	45(25.1)	
Tertiary	62(67.4)	57(65.5)	119(66.5)	
Other	2(2.2)	2(2.3)	4(2.2)	
**Occupation**				0.148
Agriculture	2(2.2)	2(2.3)	4(2.2)	
Business	28(30.4)	22(25.3)	50(27.9)	
Skilled manual	12(13.0)	10(11.5)	22(12.3)	
Blue collar	18(19.6)	8(9.2)	26(14.5)	
Professional	14(15.2)	19(21.8)	33(18.4)	
Civil servant	12(13.0)	11(12.6)	23(12.8)	
Others	6(6.5)	15(17.2)	21(11.7)	
**Frequency of alcohol intake (n = 174)**				0.903
Always	3(3.4)	4(4.7)	7(4.0)	
Often	9(10.1)	9(10.6)	18(10.3)	
Occasionally	42(47.2)	36(42.4)	78(44.8)	
Do not take	35(39.3)	36(42.4)	71(40.8)	
**Frequency of cigarette smoking (n = 166)**				0.456
Often	1(1.2)	4(4.8)	5(3.0)	
Occasionally	5(6.1)	3(3.6)	8(4.8)	
Do not take	76(92.7)	77(91.7)	153(92.2)	

Note: SD—standard deviation.

**Table 2:** Description of the sperm parameters by fertility status.

Fertility Status	Sperm Count (million)	Active Motility (%)	Total Motility (%)	Morphology (%)	Volume (mls)
**Case** (median)	6	10	20	20	2.2
IQR	14	24	36	40	1
Min	0	0	0	0	0.5
Max	76	100	309	80	5.2
**Control** (median)	46	40	60	57	2.6
IQR	26.5	19.5	15.5	20	1.5
Min	4.6	9	27	17	0.7
Max	143	85	95	80	7
**Total** (median)	26	30	50.5	50	2.5
IQR	42	31	40	42	1.1
Min	0	0	0	0	0.5
Max	143	100	309	80	7
*p*-value	<0.001	<0.001	<0.001	<0.001	0.0284

Note: *p*-value is from the Mann–Whitney test; IQR—Interquartile Range.

**Table 3:** Distribution of the antibiotics by fertility status.

Fertility Status	Ampicillin (N = 179) Median (IQR) [Range]	Metronidazole (N = 179) Median (IQR) [Range]	Tetracycline (N = 179) Median (IQR) [Range]	Chloramphenicol (N = 179) Median (IQR) [Range]	Ciprofloxacin (N = 179) Median (IQR) [Range]
**Cases (n = 87)**	0(0) [0–47]	0(19) [0–5232.67]	11(28) [0–8118]	1(3) [0–57]	11(199.47) [0–15,144.8]
**Controls (n = 92)**	0(0) [0–223]	0(0) [0–1793]	0(0) [0–6307]	0(0) [0–51]	16(146.6) [0–4454.34]
Total	0(0) [0–223]	0(0) [0–5232.67]	0(0) [0–8118]	0(0) [0–57]	14(156.28) [0–15,144.80]
*p*-value	0.8078	0.0910	0.9069	0.3587	0.6989

Note: The unit of measurement for all the antibiotics is µg/L; IQR—Inter Quartile Range. The *p*-value is from the Mann–Whitney U test.

**Table 4:** Non-parametric regression analysis estimating the marginal effect of the antibiotics on sperm parameters.

Antibiotics	Case		Control	
	Unadjusted Observed Estimate (SE) [95% CI]	Adjusted Observed Estimate (SE) [95% CI]	Unadjusted Observed Estimate (SE) [95% CI]	Adjusted Observed Estimate (SE) [95% CI]
**Sperm count**				
Ampicillin	−0.980(1.501) [−3.923–1.963]	−3.455(1.438) * [−6.276–−0.635]	−0.409(3.304) [−6.885–6.067]	−0.230(2.868) [−5.852–5.392]
Metronidazole	−0.009(0.020) [−0.049–0.031]	−0.045(0.050) [−0.144–0.053]	−0.008(0.038) [−0.084–0.067]	−0.098(1.368) [−2.781–2.584]
Tetracycline	−0.004(0.004) [−0.014–0.004]	0.004(0.017) [−0.029–0.038]	0.008(0.013) [−0.018–0.036]	0.162(0.148) [−0.128–0.453]
Chloramphenicol	−1.365(1.082) [−3.487–0.757]	−1.114(1.562) [−4.177–1.948]	0.743(1.674) [−2.538–4.024]	0.454(2.184) [−3.827–4.736]
Ciprofloxacin	0.001(0.004) [−0.007–0.009]	0.005(0.305) [−0.592–0.0603]	0.018(0.021) [−0.023–0.060]	0.026(0.028) [−0.028–0.082]
**Active motility**				
Ampicillin	1.618(0.566) ** [0.507–2.729]	−0.391(3.613) [−7.474–6.690]	−0.342(9.888) [−19.723–19.039]	−0.180(13.025) [−25.709–25.348]
Metronidazole	−0.027(0.073) [−0.171–0.116]	0.010(0.074) [−0.136–0.157]	0.004(0.048) [−0.089–0.099]	0.049(0.421) [−0.776–0.874]
Tetracycline	−0.001(0.011) [−0.023–0.020]	0.081(0.034) * [0.014–0.148]	0.015(0.013) [−0.010–0.042]	0.022(0.027) [−0.032–0.076]
Chloramphenicol	−0.719(1.23) [−3.148–1.709]	−1.140(2.68) [−6.407–4.127]	−0.021(1.07) [−2.119–2.076]	−0.415(1.05) [−2.478–1.646]
Ciprofloxacin	0.002(0.003) [−0.005–0.009]	0.006(1.519) [−2.972–2.985]	0.025(0.018) [−0.011–0.061]	−0.001(0.013) [−0.027–0.025]
**Total Motility**				
Ampicillin	1.868(2.544) [−3.119–6.855]	−0.135(7.556) [−14.945–14.674]	−0.018(8.108) [−15.910–15.874]	−0.261(6.380) [−12.767–12.245]
Metronidazole	0.025(0.075) [−0.122–0.173]	−0.173(0.594) −1.339–0.992]	0.021(0.022) [−0.022–0.065]	0.067(0.325) [−0.569–0.705]
Tetracycline	0.002(0.021) [−0.039–0.043]	0.068(0.023) ** [0.022–0.113]	0.028(0.011) * [0.006–0.050]	0.011(0.020) [−0.027–0.051]
Chloramphenicol	−2.888(1.872) [−6.559–0.781]	−3.994(1.726) * [−7.379–−0.610]	−0.351(0.860) [−2.037–1.335]	−0.609(0.896) [−2.365–1.146]
Ciprofloxacin	−0.007(0.009) [−0.025–0.010]	−0.002(0.443) [−0.872–0.867]	0.006(0.008) [−0.010–0.023]	−0.001(0.009) [−0.020–0.017]
**Morphology**				
Ampicillin	2.727(1.357) * [0.065–5.388]	3.362(3.847) [−4.177–10.902]	−1.359(3.480) [−8.181–5.462]	−0.475(6.740) [−13.687–12.735]
Metronidazole	0.046(0.044) [−0.041–0.134]	0.044(0.101) [−0.153–0.242]	0.103(0.563) [−1.001–1.207]	0.001(0.145) [−0.283–0.285]
Tetracycline	0.031(0.016) [−0.001–0.064]	0.031(0.020) [−0.008–0.071]	0.015(0.007) [−0.000–0.030]	−0.007(0.037) [−0.081–0.066]
Chloramphenicol	0.372(1.147) [−1.875–2.620]	−0.928(2.148) [−5.138–3.282]	−1.737(0.850) * [−3.403–−0.070]	−2.163(1.077) * [−4.274–−0.0522]
Ciprofloxacin	0.000(0.005) [−0.010–0.011]	0.003(2.799) [−5.483–5.490]	−0.004(0.009) [−0.023–0.014]	−0.005(0.007) [−0.020–0.008]
**Volume**				
Ampicillin	−0.037(0.083) [−0.200–0.125]	−0.036(0.112) [−0.258–0.184]	0.012(0.250) [−0.478–0.503]	0.010(0.114) [−0.214–0.235]
Metronidazole	0.003(0.002) [−0.002–0.009]	0.004(0.007) [−0.009–0.018]	0.029(0.101) [−0.169–0.229]	−0.004(0.080) [−0.163–0.153]
Tetracycline	0.000(0.001) [−0.002–0.002]	−0.000(0.001) [−0.003–0.002]	0.000(0.000) [−0.001–0.001]	−0.002(0.002) [−0.008–0.003]
Chloramphenicol	0.015(0.067) [−0.115–0.147]	0.094(0.168) [−0.235–0.424]	0.012(0.100) [−0.183–0.208]	0.023(0.116) [−0.204–0.250]
Ciprofloxacin	0.000(0.000) [−0.000–0.001]	0.000(0.122) [−0.240–0.241]	−0.000(0.001) [−0.002–0.001]	0.000(0.002) [−0.004–0.004]

Note: Effect estimates are averages of derivatives. SE is the Bootstrap standard error was used because of the small sample size. * *p* < 0.05; ** *p* < 0.01. After adjusting for multiple comparisons using the Benjamini–Hochberg false discovery rate (FDR) procedure, the associations between chloramphenicol and total motility among cases, and between chloramphenicol and sperm morphology in the control group, were no longer statistically significant.

## Data Availability

The data presented in this study are available on request from the corresponding author due to the sensitive and personal nature of the subject of the study—male infertility.

## Abbreviations

The following abbreviations are used in this manuscript:

LCMS/MS	Liquid Chromatography Mass Spectrometry/Mass Spectrometry
IQR	Inter-quartile Range
SD	Standard Deviation
SE	Standard Error

## Appendix A

**Sperm Count**

**Active Motility**

**Total Motility**

**Sperm morphology**

**Sperm volume**
